# Composition and origin of lung fluid proteome in premature infants and relationship to respiratory outcome

**DOI:** 10.1371/journal.pone.0243168

**Published:** 2020-12-10

**Authors:** Philip L. Ballard, Juan Oses-Prieto, Cheryl Chapin, Mark R. Segal, Roberta A. Ballard, Alma L. Burlingame

**Affiliations:** 1 Department of Pediatrics, University of California, San Francisco, San Francisco, California, United States of America; 2 Department of Chemistry and Pharmaceutical Chemistry, University of California, San Francisco, San Francisco, California, United States of America; 3 Department of Epidemiology and Biostatistics, University of California, San Francisco, San Francisco, California, United States of America; Hopital Robert Debre, FRANCE

## Abstract

**Background:**

Infants born at extremely low gestational age are at high risk for bronchopulmonary dysplasia and continuing lung disease. There are no early clinical biomarkers for pulmonary outcome and limited therapeutic interventions.

**Objectives:**

We performed global proteomics of premature infant tracheal aspirate (TA) and plasma to determine the composition and source of lung fluid proteins and to identify potential biomarkers of respiratory outcome.

**Methods:**

TA samples were collected from intubated infants in the TOLSURF cohort before and after nitric oxide treatment, and plasma was collected from NO CLD infants. Protein abundance was assayed by HPLC/tandem mass spectrometry and Protein Prospector software. mRNA abundance in mid-gestation fetal lung was assessed by RNA sequencing. Pulmonary morbidity was defined as a need for ventilatory support at term and during the first year.

**Results:**

Abundant TA proteins included albumin, hemoglobin, and actin-related proteins. 96 of 137 detected plasma proteins were present in TA (r = 0.69, p<0.00001). Based on lung RNAseq data, ~88% of detected TA proteins in injured infant lung are derived at least in part from lung epithelium with overrepresentation in categories of cell membrane/secretion and stress/inflammation. Comparing 37 infants at study enrollment (7–14 days) who did or did not develop persistent pulmonary morbidity, candidate biomarkers of both lung (eg., annexin A5) and plasma (eg., vitamin D-binding protein) origin were identified. Notably, levels of free hemoglobin were 2.9-fold (p = 0.03) higher in infants with pulmonary morbidity. In time course studies, hemoglobin decreased markedly in most infants after enrollment coincident with initiation of inhaled nitric oxide treatment.

**Conclusions:**

We conclude that both lung epithelium and plasma contribute to the lung fluid proteome in premature infants with lung injury. Early postnatal elevation of free hemoglobin and heme, which are both pro-oxidants, may contribute to persistent lung disease by depleting nitric oxide and increasing oxidative/nitrative stress.

## Introduction

Infants born prematurely are at risk for respiratory failure and for bronchopulmonary dysplasia (BPD), a disorder that can continue into childhood and is characterized by hypoxemia, pulmonary hypertension, wheezing and/or later asthma, requiring chronic respiratory medications and hospitalizations. The pathogenesis of BPD and associated pulmonary sequelae is not fully defined but includes lung structural and biochemical immaturity with surfactant deficiency and dysfunction, low antioxidant and immune defenses, plus exposure to hypoxia/hyperoxia and barotrauma from ventilator support; these insults damage lung epithelium, promote pulmonary edema, and elicit sterile inflammation that can be compounded by infection [1, 2]. Currently, therapeutic options for the prevention and treatment of BPD are limited and have not substantially affected the incidence of disease that remains high for the very premature infants [3].

Infants with more severe lung disease are often intubated in order to provide mechanical ventilation and airway pressure, which provides access to tracheal aspirates (TA) after saline instillation. Previous studies have analyzed constituents of lung fluid such as cytokines and surfactant components with the goal of identifying potential biomarkers for later respiratory outcome and understanding the pathogenesis of infant lung disease [4, 5]. While a number of associations between lung fluid constituents and outcome have been reported, none are in use clinically to assess risk, guide interventions or serve as a proxy for long-term outcome in clinical trials.

The continuing development of omic technologies to profile gene and protein expression and metabolite levels provides additional opportunities to explore the composition of lung fluid related to development and disease. Numerous studies have reported disease-related changes in the proteome of bronchoalveolar fluid (BALF) in adults [6–12], but information for lung fluid of infants is limited to one study [13]. In the current study, we performed a global proteomic analysis on TA samples collected from premature infants enrolled in the Trial of Late Surfactant (TOLSURF) [14, 15]. The objectives of our study were to describe the proteome of infant lung fluid, determine the origins of lung fluid proteins, and identify proteins as potential biomarkers of later respiratory status. We report quantitative data for protein levels in lung fluid and plasma, and we investigate the origin of proteins using lung transcriptomic data and proteomic data from human fetal lung and infant plasma. Comparing infants with and without lung disease persisting through the first year, we identified candidate proteins that have biological plausibility for contributing to lung injury and may serve as early biomarkers of later respiratory status.

## Materials and methods

### Study population

The Trial of Late Surfactant (TOLSURF, ClinicalTrials.gov, NCT01022580) was a blinded, randomized, sham-controlled trial performed at 25 US centers and designed to assess effects of late surfactant treatments on respiratory outcome. The trial design, infant characteristics and effects of late surfactant treatment have been reported [14]. A total of 511 extremely low gestational age (≤28 weeks) infants who required mechanical ventilation at 7–14 days were enrolled in a randomized, masked controlled trial at 25 U.S. centers. All infants received inhaled nitric oxide (iNO) beginning at enrollment, as described for the NO CLD trial (ClinicalTrials.gov, NCT00000548) [16], and either surfactant (calfactant/Infasurf) or sham installation every 1–3 days for a maximum of 5 doses while intubated. The primary outcome was survival at 36 weeks postmenstrual age without BPD as evaluated by physiological oxygen/flow reduction. 252 treated infants received surfactant and 259 controls received a sham instillation procedure. Clinical respiratory parameters were collected and respiratory severity score (RSS, FiO_2_ x mean airway pressure) was calculated. Survival without BPD did not differ between the treated and control groups at either 36 or 40 wk postmenstrual age, and there were no between-group differences in serious adverse events, comorbidities or severity of lung disease to 36 weeks. Respiratory outcomes at 1-year corrected age were assessed by quarterly questionnaires addressing use of respiratory medication, hospitalization for lung disease, and home respiratory support as reported [15].

For the current study, severe respiratory outcome was defined as a diagnosis of BPD at 36 wk and 40 wk postmenstrual age plus post-discharge respiratory support reported in 3 of 4 quarters and is designated as pulmonary morbidity (PM). This definition combines 40-wk BPD and persistent pulmonary morbidity during the first year as previously defined by Keller et al. for TOLSURF infants [15]. No/mild lung disease was defined as no BPD at 40 wk and no first-year pulmonary morbidity (No PM). Thus, the PM/No PM definition describes infants at extremes of respiratory disease. Some of the other infants who were studied over time for TA protein abundance had an intermediate respiratory outcome: ie., BPD at 40 weeks and ≤2 quarters with respiratory support or no BPD at 40 wk and post-discharge respiratory support reported in ≥1 quarter.

Biorepository plasma samples from 8 infants of the NO CLD Trial were used for proteomic assay as described for TA samples. Infants in NO CLD were similar to the TOLSURF cohort with regard to gestational age (25.3±1.3 wk) and requirement for intubation and ventilatory support at enrollment, however most were older at blood collection (41±16 d) than for TA collection in TOLSURF infants (9±2 d).

Enrolled infants from both TOLSURF and NO CLD had written, informed consent by a parent and all studies were approved by all Institutional Review Boards at the following institutions: University of Arkansas—Arkansas Children’s Hospital Little Rock, AR; Alta Bates Medical Center, Berkeley, CA; Oakland Children’s Hospital, Oakland, CA; University of California, San Francisco, San Francisco, CA; Wolfson Children’s Hospital and Shands Hospital, Jacksonville, FL; Florida Hospital for Children, Orlando, F; All Children’s Hospital, St Petersburg, FL; Northwestern Memorial Hospital, Chicago, IL; Children’s Memorial Hospital, Chicago, IL; Children’s Hospital and Clinics of Minnesota- Minneapolis, Minneapolis, MN; University of Minnesota Medical School, Minneapolis, MN; Children’s Hospital and Clinics of Minnesota—St Paul, St Paul, MN; Children’s Mercy Hospital, Kansas City, MO; Women’s and Children’s Hospital of Buffalo, Buffalo, NY; Stony Brook University Medical Center, Stony Brook, NY; Wake Forest University- Forsyth Hospital and Brenner Hospital, Winston-Salem, NC; Medical University of South Carolina, Charleston, SC; University of Tennessee Memphis- Memphis Medical Center, Memphis, TN; University of Texas Houston Health Science Center, Houston, TX; Texas Children’s Hospital, Houston, TX; University of Washington, Seattle, Seattle, WA; Children’s Hospital of Philadelphia, Philadelphia PA; University of Pennsylvania, Philadelphia PA; Case Western Reserve University, Cleveland OH; Rainbow Babies and Children’s Hospital, Cleveland OH; Brigham and Women’s Hospital, Boston MA; Children’s Hospital, Boston MA; Cedars-Siani Medical Center, Los Angeles CA; Columbus Children’s Hospital, Columbus OH; Schneider Children’s Hospital, New Hyde Park NY; Westchester Medical Center, Valhalla NY; University of Utah Hospital and Clinics, Salt Lake City, UT; Primary Children’s Medical Center, Salt Lake City, UT.

### Tracheal aspirate samples

In the TOLSURF trial, specimens of TA were collected from infants at enrollment (postnatal age 7–14 d) before treatment with surfactant or sham instillation and before starting iNO; additional samples were collected from infants before each dose of surfactant/sham. To characterize the TA proteome and investigate potential biomarkers for respiratory outcome, we selected TA samples at enrollment for 37 infants who had either PM (n = 20) or No PM (n = 17), as defined above, and were matched by study site to reduce variability in clinical management practices that might influence outcome. We used sequential TA samples from an additional 24 infants to investigate changes in protein abundance over time after enrollment. The total group of 61 infants was similar to the total TOLSURF cohort with gestational age of 25.7±1.3 wk, birth weight 734±183 g, 51% male, 43% white, 32% black, and 19% Hispanic.

The TA sampling procedure involved 2 instillations of saline into the trachea, brief ventilation, and suction to recover saline containing lung epithelial lining fluid. Samples were centrifuged at 500xg for 5 min to remove cells and the supernatant stored at -70 C in the presence of protease inhibitors.

### mRNA analysis

RNA was isolated from mid-gestation (19–23 wk gestation) specimens of human fetal lung that were collected with institutional review board approval from elective pregnancy terminations in healthy consenting women. mRNA sequencing and analysis were performed by the UCSF SABRE Functional Genomics Core using Hi-Seq 2500 machines (Illumina, San Diego, CA) as described [17].

### Fetal lung explant culture

To examine proteins in the medium of cultured lung tissue, explants of human fetal lung were prepared and cultured in serum-free Waymouth’s medium as described [18]. Fresh medium without additives was added after 24 h of culture and then collected 48 h later for proteomic analysis as described for TA samples.

### Protein analyses and statistics

Total protein in TA samples and culture medium was measured in duplicate by the Bradford assay (BioRad Laboratories, Hercules CA). Cell free hemoglobin was assayed in duplicate using Hemoglobin Human ELISA Kit (ab157707, Abcam, Burlingame, CA). For global proteomic analysis, aliquots of TA, culture medium or plasma were digested into peptides with trypsin and analyzed on a QExactive Plus mass spectrometer (Thermo Scientific, Waltham, MA) connected to a NanoAcquity^™^ Ultra Performance UPLC system (Waters, Milford MA). Peak lists were generated using PAVA in-house software [19] and searched against the human subset of the SwissProt database, using Protein Prospector [20]. The number of spectra identified as matches to peptides of a given protein were compiled, and these values (Peptide Spectral Matches, PSMs) were used for label-free quantitation of protein abundance in the samples. A relative abundance index (AI) value was calculated for each protein, which is the PSM spectral count divided by the size of the protein, divided by the total spectral counts in the sample. The mass spectrometry proteomics data have been deposited to the ProteomeXchange Consortium via the PRIDE partner repository (http://www.ebi.ac.uk/pride) with the dataset identifier PXD021966 [21].

For proteomic analysis, all the TA (or plasma samples) from each infant were run in the same batch to eliminate inter-assay variability. AI data are expressed as mean±sd for each group. Relationships between levels of proteins in TA vs plasma or culture medium were evaluated by linear regression. For analysis of protein AI in the first TA sample from infants with and without PM, we performed log transformation and Student’s t test (unadjusted p values); we also used the moderated or penalized version developed for large scale hypothesis testing situations as implemented in the R package limma, which has been demonstrated to be robust with respect to a variety of departures from normality (q values). We used p = 0.10 as a cut-off value for these exploratory studies of differential abundance. For the derived value of total hemoglobin (sum of all subunits), we used Mann-Whitney analysis. Statistical evaluation of Gene Ontology enrichment process was performed through the Gene Ontology Consortium website [22].

Initial experiments examined the intra- and inter-assay variability for each identified TA protein using 2 TA samples and 4 replicates of each sample (Supplemental S1A and S1B Fig, respectively). The coefficients of variation were dependent on the peptide count of a protein; for proteins with a peptide count >3, the coefficients of variation were 18.5% and 32.2% for intra- and inter-assay variability. Based on these data we limited quantitative comparisons of protein abundance to proteins with a mean peptide count of >3.

## Results

### TA Proteins detected by MS:MS

Studies of TA samples from 37 infants at enrollment into TOLSURF detected a total of 1839 different proteins as defined by the presence of at least 1 unique peptide in at least 1 TA sample (S1 Table). 809 of these proteins were present in ≥25% of samples, and we considered this level of occurrence in infant TA as sufficient to qualify as a TA protein. Approximately half (408) of these proteins had a mean peptide count ≥3, which was judged sufficient for quantitation of protein AI.

The 20 TA proteins with highest AI values are shown in Table 1. Albumin was the most abundant protein. Other proteins include 5 hemoglobin subunits, iron-transport/trafficking proteins *TF* and *LCN2*, 3 immune-related proteins Ig chains and *C3*, 4 actin (*ACTB*, *ACTG1*) and actin-related (*TMSB4X*, *PFN1*) proteins, and lung epithelium secreted proteins (*SERPIN1A*, *SFTPB*, *SCGB1A1*).

**Table 1:** TA proteins of highest abundance.

Protein	Gene Symbol	Peptides Detected/Sample (mean±sd)	Protein AI x 10^8^ (mean±sd)	mRNA cpm (mean±sd)
Serum albumin	*ALB*	1110±451	178.5±47	ND
Hemoglobin subunit alpha	*HBA1*	83±141	60.1±87	10±3
Hemoglobin subunit gamma-1	*HBG1*	62±96	44.0±59	28±22
Hemoglobin subunit gamma-2	*HBG2*	61±103	43.5±50	219±30
Hemoglobin subunit beta	*HBB*	50±63	37.2±46	53±4
Thymosin beta-4	*TMSB4X*	14±14	37.1±55	1525±91
Ig kappa chain C region	*IGKC*	31±9	31.6±11	ND
Actin, cytoplasmic 1	*ACTB*	114±39	31.2±8	1924±231
Actin, cytoplasmic 2	*ACTG1*	112±39	30.8±8	1942±278
Uteroglobin	*SCGB1A1*	26±18	28.7±18	ND
Serotransferrin	*TF*	174±61	25.3±6	ND
Alpha-1-antitrypsin	*SERPINA1*	101±45	24.1±8	1625±1132
Histone H4	*HIST1H4A*	23±10	22.6±8	ND
Hemoglobin subunit delta	*HBD*	27±34	20.6±26	ND
Ig gamma-1 chain C region	*IGHG1*	62±19	20.1±7	ND
Surfactant-associated protein B	*SFTPB*	76±36	20.1±7	1716±1229
Profilin-1	*PFN1*	24±7	19.1±9	331±28
Ig lambda-2 chain C regions	*IGLC2*	18±8	18.6±9	ND
Complement C3	*C3*	296±98	17.9±5	3043±833
Neutrophil gelatinase-associated lipocalin	*LCN2*	32±16	17.5±11	234±188

Protein data from TA samples of 37 infants at 7–14 days, before receiving iNO and surfactant or sham, and mRNA data from mid-trimester human fetal lung. Clinical characteristics of the infants are presented in Table 3. cpm, counts per million.

To explore which of the proteins detected in TA likely originated, at least in part, from the lung, we compared TA proteins with results by RNAseq for gene expression in late second trimester human fetal lung [17]; overall, mRNA was detected for 83% of detected TA proteins (S2 Table). By linear regression analysis of lung mRNA abundance versus TA protein AI, there was a significant slope but low positive correlation (r = 0.09, p<0.02). Thus, of the proteins in TA detected by global proteomic analysis, the great majority are produced in lung cells and may represent, at least in part, proteins secreted into lung fluid and/or released secondary to lung injury.

**Fig 1:**
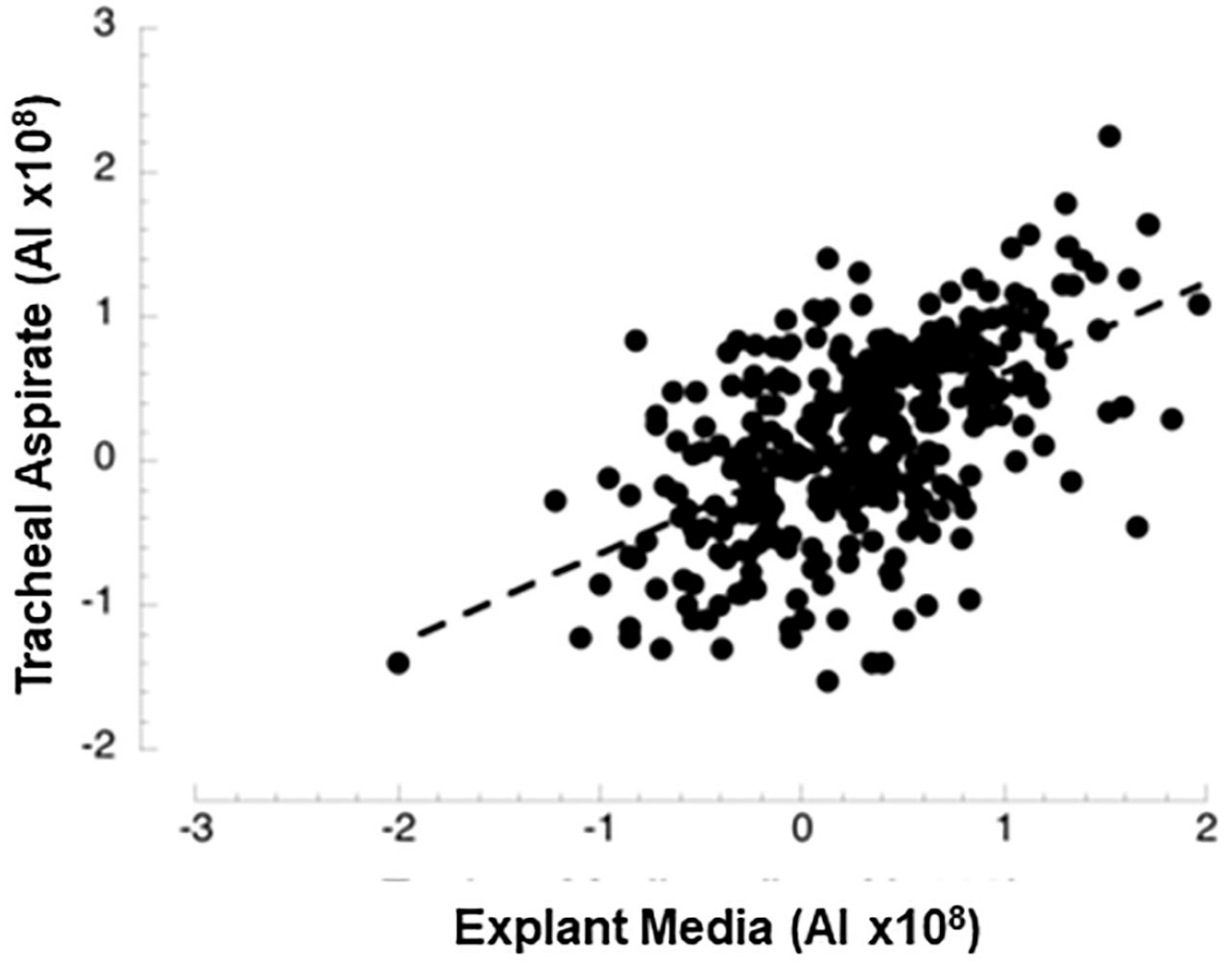
Comparison of abundance levels for proteins of TA and culture medium of human fetal lung explants. By linear regression analysis of log transformed data there is a highly significant positive correlation (r = 0.56, p<10^−30^).

In additional experiments using explants of fetal lung cultured in serum-free medium, we examined whether proteins corresponding to expressed mRNAs were released from cells [17]. Restricting the proteomic analysis to proteins with detectable lung mRNA, there were 315 proteins reliably detected and quantitated (S5 Table) in the culture medium. Protein abundance in the medium was significantly correlated with abundance in TA (Fig 1). Some apparent outlier proteins were observed including 4 hemoglobin subunits with relatively high TA abundance and 3 proteins with low TA abundance relative to the medium: heme binding protein 2, tissue inhibitor metalloprotease 1 and a histone.

Analysis of TA proteins by Gene Ontology revealed highly significant overrepresentations of specific groups of proteins by both cellular component and biological process categories with enrichment ranging between 2- and 9-fold as summarized in Table 2. Highest categories of localization were for proteins of focal adhesions in the extracellular matrix, membrane rafts, cytoskeleton, and secreted/extracellular organelles. By function, enriched categories included proteins primarily related to secretion, protein processing and inflammation with a 9-fold enrichment of proteins related to neutrophil activation and function. In general, these findings are consistent with both normal lung epithelial cell function (e.g., secretion of surfactant) and inflammatory plus cell damage responses to lung injury in the immature lung [23]. A full listing of gene ontology results is given in S3 and S4 Tables.

**Table 2:** Cellular localization and biological process of TA proteins by gene ontology.

GO Cellular Component	GO Proteins	TA Proteins	Expected Proteins	Fold Enrichment
Focal adhesion/cell-substrate junction	404	104	12	8.5
Extracellular exosome/vesicle/organelle	2109	488	64	7.6
Secretory granule/vesicle	911	183	28	6.7
Lysosome	670	94	20	4.6
Actin cytoskeleton	490	73	15	4.9
Membrane raft/microdomain	304	41	9	4.4
Intrinsic/integral component of membrane	5568	114	169	0.67
**GO Biological Process**				
Neutrophil function	496	141	15	9.4
Protein folding	182	31	6	8.2
Exocytosis/secretion	881	177	27	6.8
Inflammatory response	343	36	10	3.7
Response to cytokine	687	65	21	3.4
Response to stress	1349	105	41	3.3
Immune system/response/activation	1258	118	38	3.1
Protein transport/localization	966	71	29	2.9
Regulation of protein metabolic process	1014	79	31	2.9
Transcription-related	2564	37	78	0.47

GO categories for TA proteins with detectable mRNA in fetal lung. p values with Bonferonni correction ranged from 1.0E-79 to 1.0E-6 except for the 2 underrepresented categories (intrinsic/integral component of membrane and transcription-related) with p<0.005.

### Plasma proteomics

**Fig 2:**
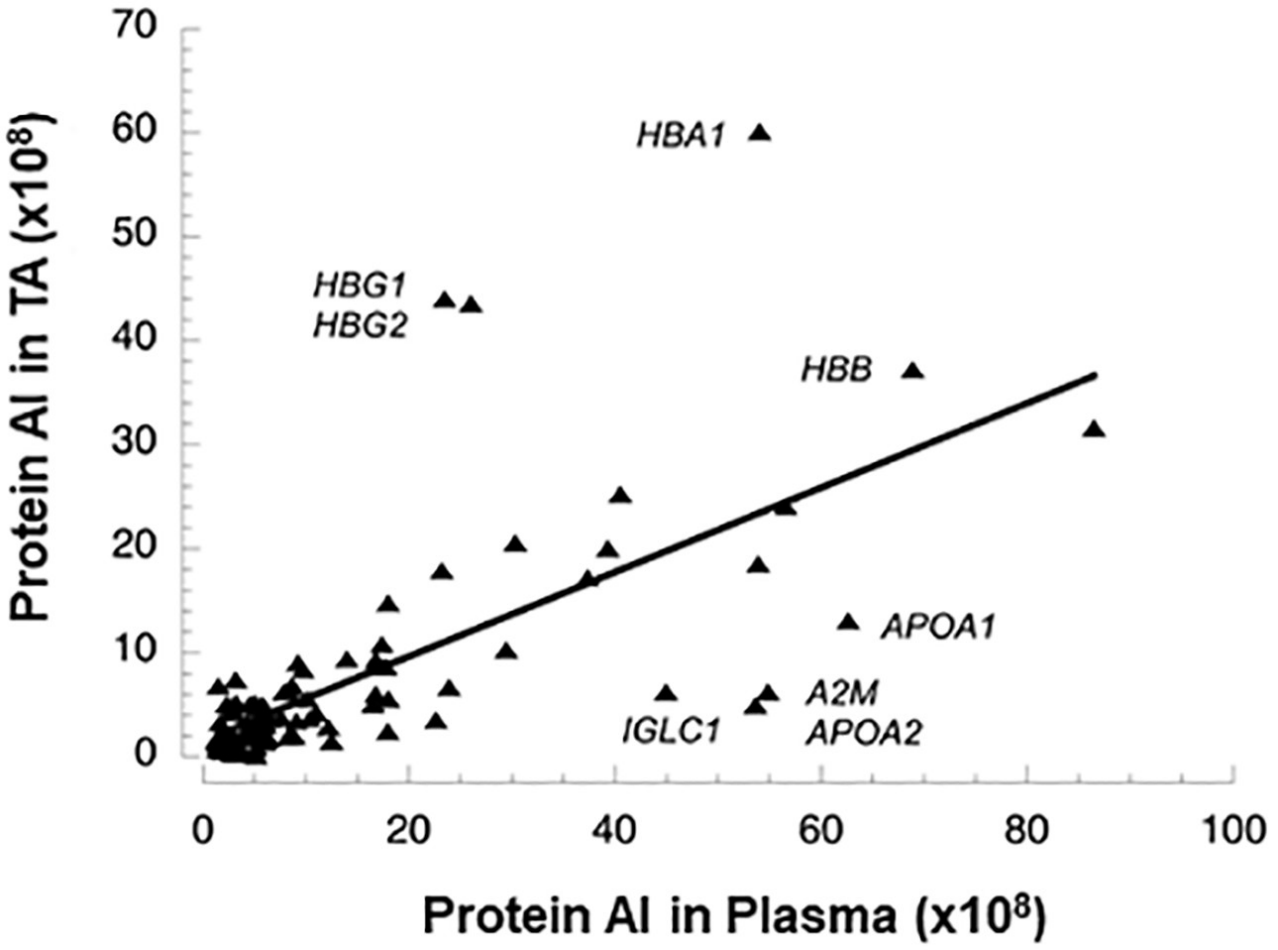
Comparison of abundance levels for proteins present in both TA and plasma of premature infants. By regression analysis r = 0.69 (p<0.00001) excluding albumin as an outlier, which had AI values of 179 and 315 for TA and plasma, respectively. Gene symbols are shown for some outlier proteins.

To directly investigate the contribution of circulating proteins to the infant TA proteome, we performed proteomic analysis of plasma using banked samples from intubated premature infants in the NO CLD trial (n = 8, mean gestational age 25.3 wk). A total of 435 proteins were detected in at least 25% of the plasma samples (S6 Table). Of these, 137 proteins were consistently present with a mean peptide count of ≥3, providing a reliable value for AI, and of these, 96 (70%) were also consistently detected in TA samples. For these proteins, there was a close association between AI levels in TA and plasma (Fig 2, r = 0.69, p<0.00001) excluding albumin, which was many-fold more abundant than other proteins. The median ratio of AI for all proteins in TA vs plasma was 0.52 with some outlier values: hemoglobin subunits alpha and gamma 1 and 2 had higher TA:plasma ratios (2.2 to 4.5), and Ig lambda chain C, alpha2 macroglobulin and apolipoproteins A1 and A2 had TA:plasma ratios <0.2. The TA abundance of the 11 outlier proteins was not related to protein molecular weight, which could affect trans-endothelial passage, nor was there a correlation between molecular weight and TA:plasma ratio for all proteins (r = 0.1, p = 0.33). The finding that 96 of 809 proteins detected in TA are also found in plasma suggests that influx of proteins into air spaces from the circulation can contribute up to ~12% of detected lung fluid proteins. By relative abundance, 15 of the top 20 most abundant proteins of TA (Table 1) are plasma proteins.

### TA proteins and respiratory outcome

One objective of this study was to identify TA proteins that differed in abundance by two extremes of respiratory outcome. The 37 infants used for study of TA protein composition and origin were selected by matching infants by site. 20 PM infants and 17 No PM infants that had TA samples at enrollment were available for study; key demographics are shown in Table 3. All infants were at high risk for BPD based on TOLSURF entry criteria. Mean gestational ages were ~26 weeks with mean birth weights of 685 and 748 g, respectively. The PM group had somewhat more males and Caucasians and a higher RSS score than the NO PM infants, but these differences were not significant; however, the number of blood transfusions was 2-fold higher (p<0.01) in PM infants.

**Table 3:** Characteristics of extreme outcome infants in study of TA proteins.

	No PM	PM
N	17	20
Gestational age (wk)	25.9±1.2	25.5±1.4
Birth weight (g)	748±199	685±177
Male/female	6/11	13/7
Race (Caucasian/AA/Hispanic/Other)	5/6/5/1	11/6/2/1
Age enrollment/TA collection (days)	9.3±2.7	8.9±2.1
Number of clinical sites	12	13
Late surfactant therapy	9 (52.9%)	13 (65%)
RSS at TA collection	2.6±0.9	3.3±1.8
Blood transfusions (total number)	6.1±5.7	11.5±6.2^+^
Patent ductus arteriosus—treated	5 (29.4%)	2 (10%)
Culture-positive sepsis	2 (11.8%)	0 (0%)
Severe pulmonary hemorrhage	1 (5.9%)	1 (5%)
Survive without BPD36wk (yes/no)	16/1	0/20
Survive without BPD40wk (yes/no)	17/0	0/20
Pulmonary morbidity first year (none/severe)*	17/0	0/20

Data for patent ductus arteriosus, sepsis and pulmonary hemorrhage are for the interval between birth and enrollment.

*Severe is persistent pulmonary morbidity defined as respiratory support (medication, home respiratory support, hospitalization) in ≥3 of 4 quarters by questionnaire during the first year, and none is no reports of respiratory support during first year [15].

^+^ p<0.01 by two-sided unpaired t test; AA, African American; RSS, respiratory severity score (FiO_2_ x MAP).

**Fig 3:**
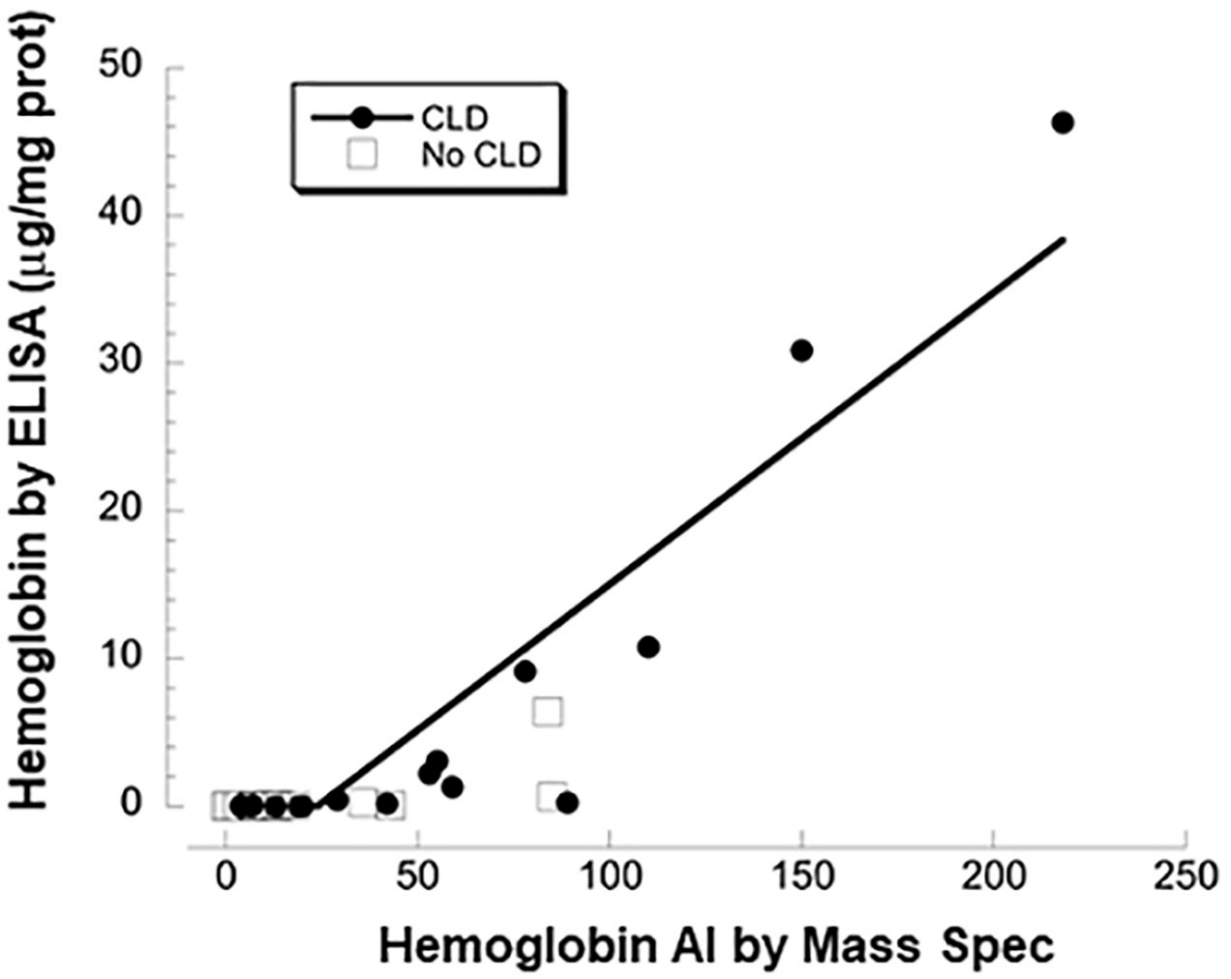
Comparison of abundance levels for total hemoglobin of TA by MS and ELISA assay. Solid circles are infants with PM and open squares are No PM. n = 34, r = 34, p = 0.00001 by linear regression. Except for lower values, results by immunoassay correlate with MS results.

Table 4 shows 20 TA proteins selected for apparent difference (unadjusted p<0.10) in abundance by respiratory outcome; 11 proteins were decreased (fold range 0.44–0.83) and 9 were increased (fold range 1.4–4.4) in PM vs No PM infants. A full list of protein abundance by outcome is shown in S7 Table. mRNA was not detected in fetal lung for 7 of the proteins, consistent with plasma as the source. Due to the limited number of infants studied and the large number of proteins assayed, none of the differences reached q<0.10 by false discovery rate. Nevertheless, these are candidate proteins of interest for association with respiratory outcome. Of particular interest, all detected subunits of hemoglobin were higher in PM infants (2.6- to 4.4-fold), as was the derived value for total hemoglobin (2.9-fold, p = 0.029), whereas levels were ~1.0 for the major plasma proteins albumin and total immunoglobulins. For samples with higher levels of hemoglobin, there was a strong, positive correlation between levels determined by MS:MS versus immunoassay (Fig 3).

**Table 4:** Selected proteins and relative abundance in TA of PM vs No PM infants.

Protein	PM vs No PM		Fetal Lung Expression	
	Fold	AI	Protein	mRNA
Vitamin D-binding protein	0.77	10.7	1.2	<2
Gamma-enolase	0.71	2.1	2.3	192
Annexin A5	0.77	8.7	11.5	880
Complement C3	0.83	18.1	41.7	3043
Ubiquitin-like modifier-activating enzyme 1	0.62	0.5	<0.05	<2
Malate dehydrogenase, mitochondrial	0.48	0.6	2.6	113
Heterogeneous nuclear ribonucleoprotein A1	0.59	0.8	2.2	1179
Alpha-actinin-2	0.72	1.2	1.3	<2
Dipeptidyl peptidase 1	0.63	1.2	0.6	389
Tetranectin	0.63	1.8	2.2	<2
Purine nucleoside phosphorylase	0.52	1.5	<0.05	77
Vitamin K-dependent protein S	0.56	0.6	0.6	188
Rab GDP dissociation inhibitor beta	0.79	3.2	2.6	388
Alpha-1-acid glycoprotein 2	0.78	6.6	0.5	20
Beta-hexosaminidase subunit beta	0.58	0.5	0.4	118
Collagen alpha-1(VI) chain	0.53	0.6	1.5	145
Heat shock 70 kDa protein 6	1.4	1.6	1.2	61
Putative heat shock 70 kDa protein 7	1.55	1.7	1.8	9
Ras-related protein Rab-1B	1.96	1.1	<0.05	39
Histone H2A type 2-C	2.82	2.3	<0.05	<2
Hemoglobin subunit gamma-2	3.91	42.8	52	219
Hemoglobin subunit epsilon	2.77	5.1	<0.05	<2
Hemoglobin subunit gamma-1	2.85	43.3	51.4	28
Hemoglobin subunit beta	2.47	36.5	13.3	53
Hemoglobin subunit delta	2.36	20.2	<0.05	<2
Hemoglobin subunit zeta	3.45	2	1.9	<2
Hemoglobin subunit alpha	2.41	59.9	20.2	10
Total hemoglobin*	2.92	206.7	138.8	310

TA proteins with p value <0.10 (unpaired t test) comparing levels at enrollment of 37 infants with extreme respiratory outcomes. Levels of expression in lung are expressed as AI (protein abundance in explant medium) and mRNA as counts per million (cpm).

*Calculated as sum of all subunits; p = 0.029 by Mann Whitney analysis.

### TA time course

**Fig 4:**
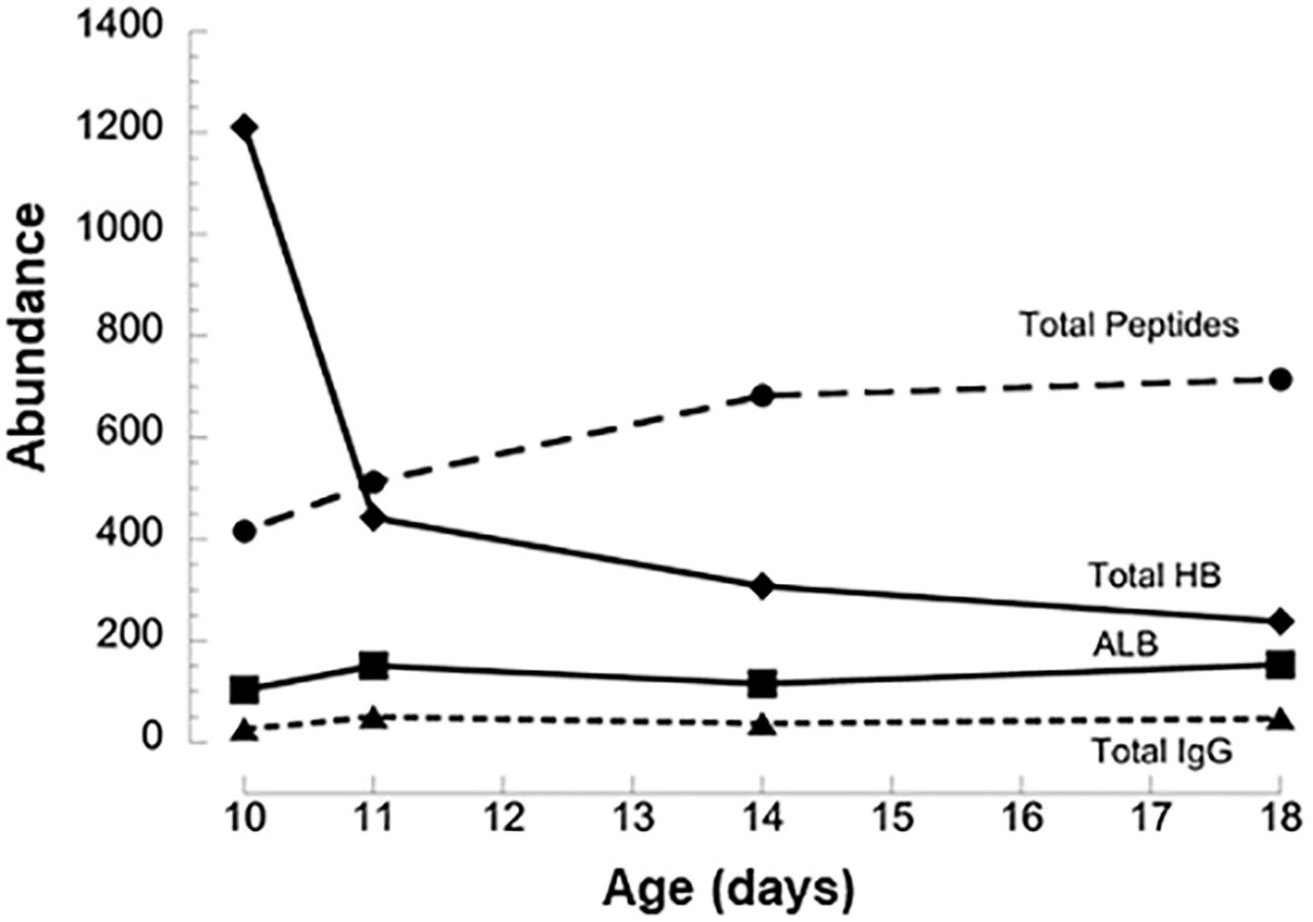
Time course for TA levels of total peptides, total hemoglobin, albumin and total IgG. Example of one infant with TA samples collected between 10 and 18 d postnatal age, showing decreases over time in hemoglobin, but not other major plasma proteins. Values are AIx10^8^ except for total peptide.

We analyzed protein abundance in additional, consecutive TA samples collected for a separate group of 24 infants with at least 3 longitudinal TA samples (7 PM, 9 No PM, 8 with intermediate outcome) between 7 and 21 days (3–5 TAs per infant over 8±2 days). For the 13 non-hemoglobin proteins that are candidates for differential abundance by respiratory outcome (Table 4), there was no consistent temporal change in AI levels with sequential samples over time. Similarly, serum albumin was present in every TA sample with a relatively narrow AI range (99–309) and no consistent temporal pattern. By contrast, the level of total hemoglobin decreased over time after trial enrollment in 17 of 21 infants with detectable hemoglobin to a mean level in the final TA sample of 15±10% of the first value; there was no consistent change in hemoglobin in 4 infants (3 PM infants and 1 intermediate outcome infant—last sample mean 99% of first sample). The decrease in total hemoglobin with time was similar for No PM and PM infants (14±15% vs 17±10% of the first value, respectively). Fig 4 illustrates decreasing total hemoglobin compared to albumin, total IgG and total peptide count for a PM infant with an initial high level of total hemoglobin.

## Discussion

In this study, we used global proteomic analysis to provide new information related to TA proteins and their relative abundance in intubated premature infants. We also determined the proteome of premature infant plasma and examined both gene expression and presence of secreted proteins in human fetal lung. Assessing these complementary data, we report, to our knowledge, the first description of the origin of major proteins in TA of the premature infant. We conclude that up to 88% of the detected proteins in TA of these infants are likely derived primarily from lung epithelial cells via secretion and/or release secondary to injury to the extracellular matrix, cell membrane and cytoskeleton. Other TA proteins are primarily derived from plasma, with a strong correlation between relative abundance in plasma and TA. When the origin of TA protein is considered in terms of bulk amount, plasma contributes ~28% of total protein with serum albumin and free hemoglobin as major contributors. In comparison of the proteomes of two infant cohorts with extreme respiratory outcomes, candidate protein biomarkers for PM of both lung and plasma origin were identified. The most notable of these was cell-free hemoglobin, which was elevated 2.9-fold in PM vs No PM infants at study enrollment.

Analysis of the infant TA proteome data by gene ontology revealed overrepresentation of proteins consistent with injury to the lung epithelium and inflammation. We did not have access to TA from control premature infants without lung disease, who are not intubated, but proteome data by a similar shotgun proteomic approach) are available for normal adult BALF [12]. Various families of proteins with relatively high abundance in infant TA were not detected in normal adult lung fluid: collagens, actins, heat shock proteins, histones, keratins, tubulins and subunits of fetal and embryonic hemoglobin. These differences between premature infant and adult lung could reflect a response to lung injury and/or developmental changes in expression levels.

Proteomic analyses of human lung fluid obtained by bronchopulmonary lavage has been reported for a variety of adult lung diseases including COPD [6], pulmonary sarcoidosis [7], idiopathic pneumonia syndrome [8], idiopathic pulmonary fibrosis [9], acute respiratory distress syndrome [10], ventilator-associated pneumonia [11] and HIV [12]. Most relevant to our study, in lung injury due to acute respiratory distress syndrome, Bhargava et al. [10] reported that non-survivors showed overrepresentation by gene ontology of proteins related to carbohydrate catabolism, disorganization of actin filaments and collagen biosynthesis. In a global proteomic study of adults with acute lung injury, Nguyen et al [12] found differential expression of nearly half of all detected proteins compared with normal controls.

To our knowledge, there has been only one previous report of proteomic analysis of infant tracheal aspirates. Magagnotti et al. [13] reported lower abundance of 2 calcium-binding proteins (*CAPS*, *CIB1*) and the chloride channel protein (*CLIC1*) for 6 of 12 infants who developed severe vs mild BPD. These proteins are expressed in fetal lung [17] and were detected in our proteomic study, however abundance in TA was not different by respiratory outcome.

The plasma proteome for adults and infants [24] has been previously described, but, to our knowledge, we provide the first comparison of infant plasma and lung fluid protein profile and evidence for the close proportional abundance in the two tissue compartments. This finding indicates that most plasma proteins, irrespective of their size, pass the endothelial/epithelial barrier and accumulate in lung fluid in the injured infant lung similar to the observation in adults with acute respiratory distress syndrome [25].

This study has limitations primarily related to methodology and the infant cohort. Proteomics by HPLC MS:MS provides a global survey but does not have the sensitivity of some other approaches such as immunoassay, which has documented the presence of numerous cytokines and chemokines in infant TA that we did not routinely detect [4]. Because plasma samples were not collected in TOLSURF, we used plasma from a separate cohort of infants (NO CLD) of similar demographics and severity of lung disease, however the plasma samples were collected later in the first month compared to the TA samples; future confirmation of relative abundance of proteins in TA versus plasma should involve matched TA:plasma samples from infants. Importantly, our findings are for infants with lung injury, and it is known from lamb studies that ventilator-induced lung injury results in a rapid and large change in protein composition of lung tissue, including extracellular proteins of epithelial origin [26]. Also, we compared TA proteins with proteins secreted from cultured fetal lung, which was not exposed to the insults associated with postnatal lung injury. In the biomarker component of our study we were limited in terms of the number of available infants with TA samples who had extreme respiratory outcomes. Because of this limitation we could not analyze our results by multivariate modeling to account for clinical predictors of lung disease, and thus our results for candidate proteins should be viewed as preliminary and will require validation in another, larger cohort.

The level of plasma free hemoglobin in healthy individuals is undetectable or low (<15 mg/dl). Levels can be many-fold higher with extra-corporeal membrane oxygenation and hemolytic anemias, but do not usually achieve the concentrations that we observed in plasma of infants with lung disease (~50% of albumin or ~1000 mg/dl). We also found high abundance of hemoglobin in TA, at levels similar those for albumin. By contrast, the level of hemoglobin alpha/beta was <5% of albumin in lung fluid of adults with acute lung injury [11, 12]. Thus, high levels of hemoglobin in lung fluid and plasma with lung injury may be unique to the premature infant, although further studies on this topic are needed.

The second observation regarding TA hemoglobin was the association of increased levels with later pulmonary morbidity. Of interest, Pereira-Fantini et al. [27] recently reported on changes in the plasma proteome in newborn premature lambs with exposure to 60 minutes of ventilator-induced lung injury. Similar to our results, three hemoglobins had the highest fold-increase (>2-fold) among 14 differentially abundant proteins of 125 total identified plasma proteins.

We have considered possible sources of increased cell-free hemoglobin in infant lung fluid: 1) intra-pulmonary bleeding and hemolysis; 2) increased synthesis/release of selected hemoglobin subunits (alpha, beta, gamma 1/2) from lung epithelial cells; 3) influx of cell-free plasma hemoglobin derived from blood transfusions; and 4) intrapulmonary hemolysis secondary to endothelium injury/dysfunction. Diagnosis of severe pulmonary hemorrhage was low (5–6%) and not different between outcome groups, however no clinical data are available for milder forms of hemorrhage. The second possibility (synthesis in lung) is supported by the finding of hemoglobin expression in human fetal lung (Table 4) and in lung type 2 cells of both fetal and adult human [23, 28] and by the report of increased hemoglobin gene expression and protein accumulation in rats exposed to hypoxia, which is a component of infant lung disease [29]. Cell-free hemoglobin and iron derived from transfused packed red cells, particularly with longer storage, may contribute to long-term lung disease based on the greater number of transfusions in PM vs No PM infants (Table 3) and on previous discussion [30]. The fourth possibility, hemolysis in the pulmonary circulation (and perhaps systemically) secondary to lung injury/oxidative stress, is supported by the observation of increased plasma hemoglobin subunits in lambs with ventilator-induced lung injury [27]. The finding of increased free hemoglobin with lung injury in two species supports further study of free hemoglobin (plasma and TA) as a potential early biomarker and monitoring approach for the severity of lung injury in infants.

Increased cell-free hemoglobin in lung fluid of infants, regardless of the sources, has biological plausibility for contributing to lung injury and affecting respiratory outcome. As an abundant protein, it could contribute to protein inhibition of surfactant activity, particularly when surfactant is deficient [5]. Second, as recently reviewed [31], cell-free hemoglobin is a pro-oxidant that would promote oxidative stress systemically and within airspaces through reactions with peroxides and nitric oxide (NO). Consumption of biologically available NO affects both vascular tone (eg., pulmonary arterial hypertension) and angiogenesis/repair processes after lung injury [32]. In studies with mice, tracheal instillation of cell free hemoglobin increased alveolar-capillary permeability, inflammatory cell influx and cytokine production more than albumin instillation [33]. Third, oxidation of hemoglobin via reaction with NO releases free heme that is toxic to membrane lipids and lipoproteins, inducing inflammation, cell injury and possibly surfactant dysfunction. Reduced amount of hemopexin, a heme scavenger protein, as observed in PM infants could also increase heme toxicity. This model of cell-free hemoglobin/heme toxicity in the lung is supported by known adverse effects of intravascular hemolysis in liver, spleen, kidney and vessels that are associated with sickle cell disease, malaria, blood transfusions cardiopulmonary bypass and sepsis [31].

The reason for the observed decrease in TA hemoglobin, but not in other candidate biomarker proteins, for most infants in our time course studies is unknown. Because albumin and total IgG did not decrease with time, the decline in hemoglobin levels in lung fluid cannot be explained by improving overall barrier function. In general, lung disease does not improve in these infants during this time and there were no consistent changes in respiratory support or medications with one exception: iNO was started in all infants after collection of the first TA sample. We speculate that NO therapy may reduce lung injury secondary to hemolysis similar to the observed benefit of iNO for acute kidney injury associated with bypass [34].

If a role of cell-free hemoglobin in the pathogenesis of PM in infants is confirmed by further studies, some possible targeted therapeutic approaches can be considered. In addition to iNO treatment, which reduces BPD in infants of maternally-identified African-American women [35], therapies with scavenger proteins such as haptoglobin and hemopexin could reduce the concentration of hemoglobin and heme, respectively [31], One approach to address oxidative stress is superoxide dismutase, a therapy that has been tested in infants [36].

Another candidate protein of particular interest with regard to pathogenesis of BPD is vitamin D binding protein (*GC*), which was lower in TA of PM infants. Vitamin D binding protein is the major transporter for vitamin D, a scavenger of actin in damaged cells as occur in the injured lung [37], implicated as a positive regulator of lung development [38], and impacts the immune response by enhancing complement-mediated neutrophil chemotaxis. Altered vitamin D binding protein has been associated with pulmonary sarcoidosis, acute respiratory distress syndrome, chronic obstructive pulmonary disease and asthma in adults [39, 40], and there is one report for association with BPD [41].

In summary, we provide new information on the protein composition of lung fluid in premature infants with lung disease, combining global proteomic and transcriptomic analyses and using a cohort of infants with extensive clinical data. The findings allow assignment of the origin of most lung fluid proteins as plasma or lung cells and also identify candidate biomarker proteins for continuing infant lung disease through the first year of life. We observed elevated hemoglobin subunits in PM infants and discuss a possible causal role for these proteins in the pathogenesis of lung disease.

## Supporting information

S1 Fig Assay variability based on peptide count. A, Intra-assay. B, Inter-assay. The coefficient of variability (CV) from replicate experiments improves with increasing peptide count. A peptide count >3 was used for all analyses.
(TIF)

S1 Table Proteins detected in infant tracheal aspirate, sorted by AI. (XLSX)

S2 Table Abundance of fetal lung mRNA for TA proteins, sorted by mean RNA cpm. Data by RNAseq for human fetal lung. ND, not detected, mean cpm <4.
(XLSX)

S3 Table Cellular localization of TA proteins by gene ontology. GO categories for TA proteins with detectable mRNA in fetal lung. p values <0.05 with Bonferonni correction.
(XLSX)

S4 Table Biological process of TA proteins by gene ontology. GO categories for TA proteins with detectable mRNA in fetal lung. p values <0.05 with Bonferonni correction.
(XLSX)

S5 Table Proteins detected in medium of human fetal lung explants, sorted by AI. Data for culture medium from explants of 3 specimens of human fetal lung.
(XLSX)

S6 Table Proteins detected in infant plasma, sorted by peptide count. Data from plasma of 8 premature infants.
(XLSX)

S7 Table TA protein abundance for PM vs No PM infants, sorted by fold difference. (XLSX)
