# Supplementary figures and images for: Composition and origin of lung fluid proteome in premature infants and relationship to respiratory outcome
Source: PLoS One. 2020 Dec 10;15(12):e0243168. doi: 10.1371/journal.pone.0243168 (PMC7728257; doi:10.1371/journal.pone.0243168)

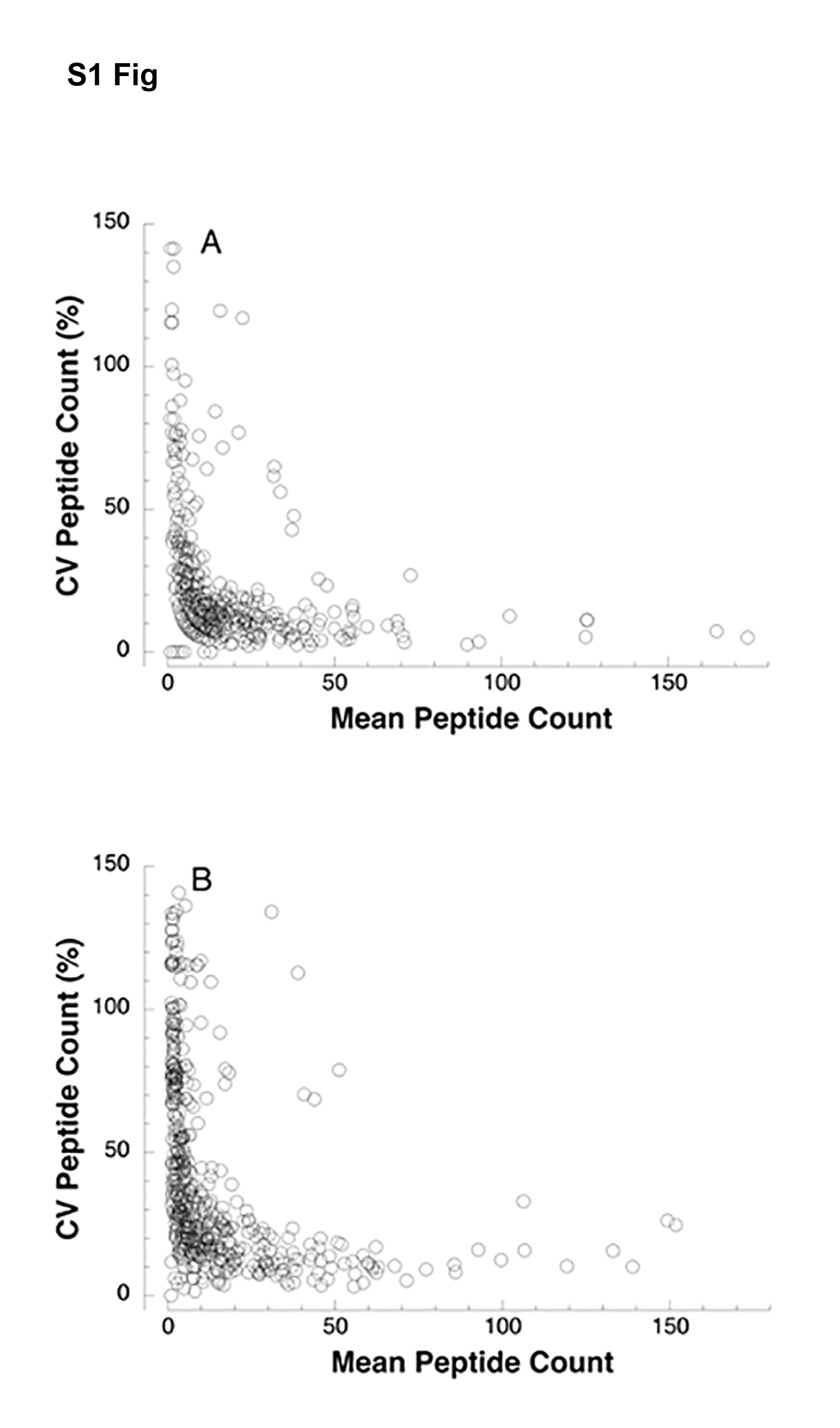

Supplement: S1 Fig — A, Intra-assay. B, Inter-assay. The coefficient of variability (CV) from replicate experiments improves with increasing peptide count. A peptide count >3 was used for all analyses. (TIF) [file pone.0243168.s001.tif]
